# Supplementary material for: The Binding of CSL Proteins to Either Co-Activators or Co-Repressors Protects from Proteasomal Degradation Induced by MAPK-Dependent Phosphorylation
Source: Int J Mol Sci. 2022 Oct 15;23(20):12336. doi: 10.3390/ijms232012336 (PMC9604145; doi:10.3390/ijms232012336)
Supplement: Supplementary file 1 [file ijms-23-12336-s001.zip › ijms-1968898-supplementary.pdf]

Supplementary Material

Figure S1. Quantification of Su(H) protein amounts upon blocking proteasomal activity

Figure S2. Mutation in individual lysine residues have little impact on the stability of Su(H)

Figure S3. Original Western blots used for the quantification of $Su(H)^{wt\text{-}myc}$ and $Su(H)^{LLL\text{-}myc}$ amounts in $RBPJ^{KO}$ HeLa cells

Figure S4. Original Western blots used for the quantification of $Su(H)^{wt\text{-}myc}$ and $Su(H)^{LLL\text{-}myc}$ amounts in $RBPJ^{KO}$ HeLa cells in the presence of H

Figure S5. Original Western blots used for the quantification of $Su(H)^{wt\text{-}myc}$ and $Su(H)^{LLL\text{-}myc}$ amounts in $RBPJ^{KO}$ HeLa cells in the presence of Notch ICN^{RAMANK}

Figure S6. Original Western blots used for the quantification of wild type human RBPJ and murine Rbpj amounts, respectively, in $RBPJ^{KO}$ HeLa cells

Figure S7. Protein-interaction analyses of Rbpj mutant variants

Figure S8. Original Western blots used for the quantification of mutant murine $Rbpj^{FAL}$ and $Rbpj^{EEFAL}$ amounts, respectively, in $RBPJ^{KO}$ HeLa cells

Table S1. List of primers used for mutagenesis

Figure S1. Quantification of Su(H) protein amounts upon blocking proteasomal activity

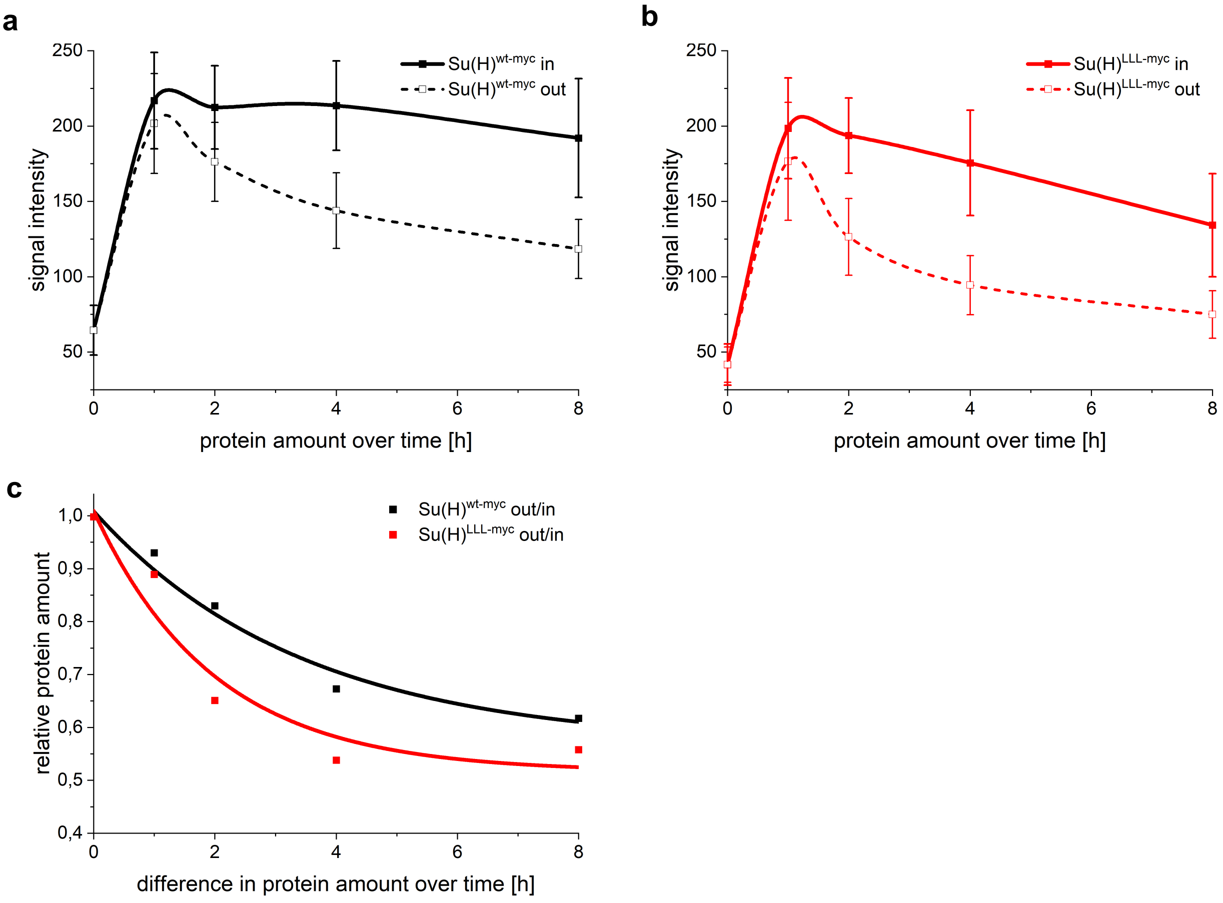

Su(H)$^{wt\text{-}myc}$ **(a)** and Su(H)$^{LLL\text{-}myc}$ **(b)** protein levels over time were quantified within the ptc-Gal4 overexpression domain (*in*, solid line) and in the direct neighbourhood (*out*, dashed line) in the ptc::DTS5-wing discs. The lower peak at 1 h *out* compared to *in* indicates protein degradation to occur immediately after heat induction of the protein in the presence of proteasomal activity. **(c)** The percental decline of Su(H)$^{wt\text{-}myc}$ protein (black line) and Su(H)$^{LLL\text{-}myc}$ protein (red line), respectively, in the presence of proteasomal activity is given, i.e. the relation of protein amount *out* to *in* over time. The increased instability of Su(H)$^{LLL\text{-}myc}$ is clearly visible by the faster decline of the curve (red line).

Grey levels were recorded at twelve points each within (*in*) and directly adjacent outside (*out*) the ptc-Gal4 overexpression domain in the presumptive wing blade area using Image J. Each value represents the average measurements from five discs; SD is indicated.

Figure S2. Mutation in individual lysine residues have little impact on the stability of Su(H)

a

```
hRBPJ   27 PPKRLTREAM RNYLKER   43
mRbpj   53 PPKRLTREAM RNYLKER   69
Su(H)  100 EEKKLTRDAM EKYMRER  117

           .......... ....

hRBPJ  122 YCTAKTLYIS DSDKRKH  138
mRbpj  148 YCTAKTLYIS DSDKRKH  164
Su(H)  196 YCAAKTLFIS DSDKRKH  212

           .......... .......

hRBPJ  267 VDKQTALLDA DDPVSQLHKC 286
mRbpj  293 VDKQTALLDA DDPVSQLHKC 312
Su(H)  341 VDKQMALLEA DDPVSQLHKC 360

hRBPJ  287 AFYLKDTERM YLCLSQERII 306
mRbpj  313 AFYLKDTERM YLCLSQERII 332
Su(H)  361 AFYMKDTDRM YLCLSQERII 380

hRBPJ  307 QFQATPCPKE PNKEMINDGA 326
mRbpj  333 QFQATPCPKE QNKEMINDGA 352
Su(H)  381 QFQATPCPKE PNKEMINDGA 400
```

b

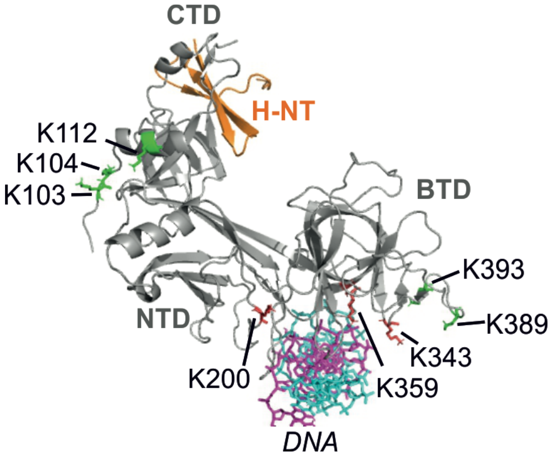

c

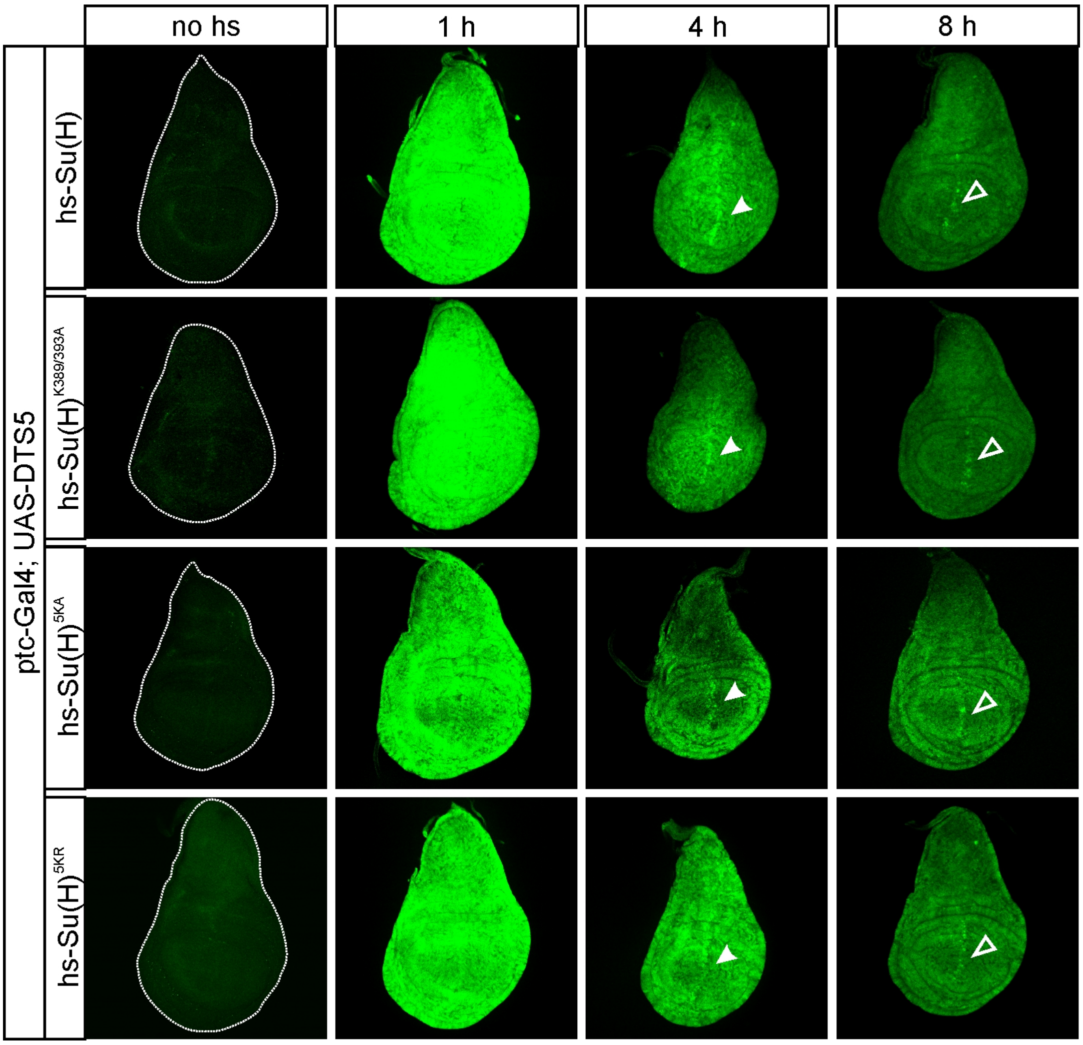

Legend to Supplemental Figure S2

Mutation in individual lysine residues have little impact on the stability of Su(H)

(a) Sequence alignment of CSL peptides from human (hRBPJ, NP_005340.2), mouse (mRbpj, NP033061.3) and fly Su(H) (NP_476868.1); only relevant parts are shown, sequence numbers indicated. Identical amino acids are highlighted in light blue, similar ones in red. Lysine residues, changed in the course of the examination, are shown in bold and yellow. In *Drosophila* Su(H), five lysine residues are predicted to be ubiquitinated, K103, K104 with high confidence, K112 and K393 with low and K389 with medium confidence (Radivojac, et al. **2011**. DOI: 10.1002/prot.22555 [56]).

(b) Ribbon diagram of the Su(H)-H repressor complex bound on the DNA based on X-ray structures (PDB-ID 5E24). The structural domains of Su(H), the N-terminal domain (NTD), the beta-trefoil domain (BTD) and the C-terminal domain (CTD) are indicated, as is the Su(H)-binding domain of H depicted in amber (H-NT). Lysine residues mutated in the course of this work are highlighted.

(c) DTS5-experiment performed on the double mutant hs-Su(H)$^{K389/393A}$, and the two quintuple mutants hs-Su(H)^{5KA} (K103,104,112,389,393A) and hs-Su(H)^{5KR} (K200,343,359,389,393R). Stability of the respective mutant protein, induced by a 30 min heat pulse, monitored over time. Local overexpression of DTS5 (ptc-Gal5; UAS-DTS5) results in proteasomal dysfunction along the antero-posterior boundary, where protein stabilization is observed (arrowheads). Su(H) protein degradation of any mutant is similar to the wildtype.

Figure S3. Original Western blots used for the quantification of Su(H)$^{wt\text{-}myc}$ and Su(H)$^{LLL\text{-}myc}$ amounts in RBPJ^{KO} HeLa cells.

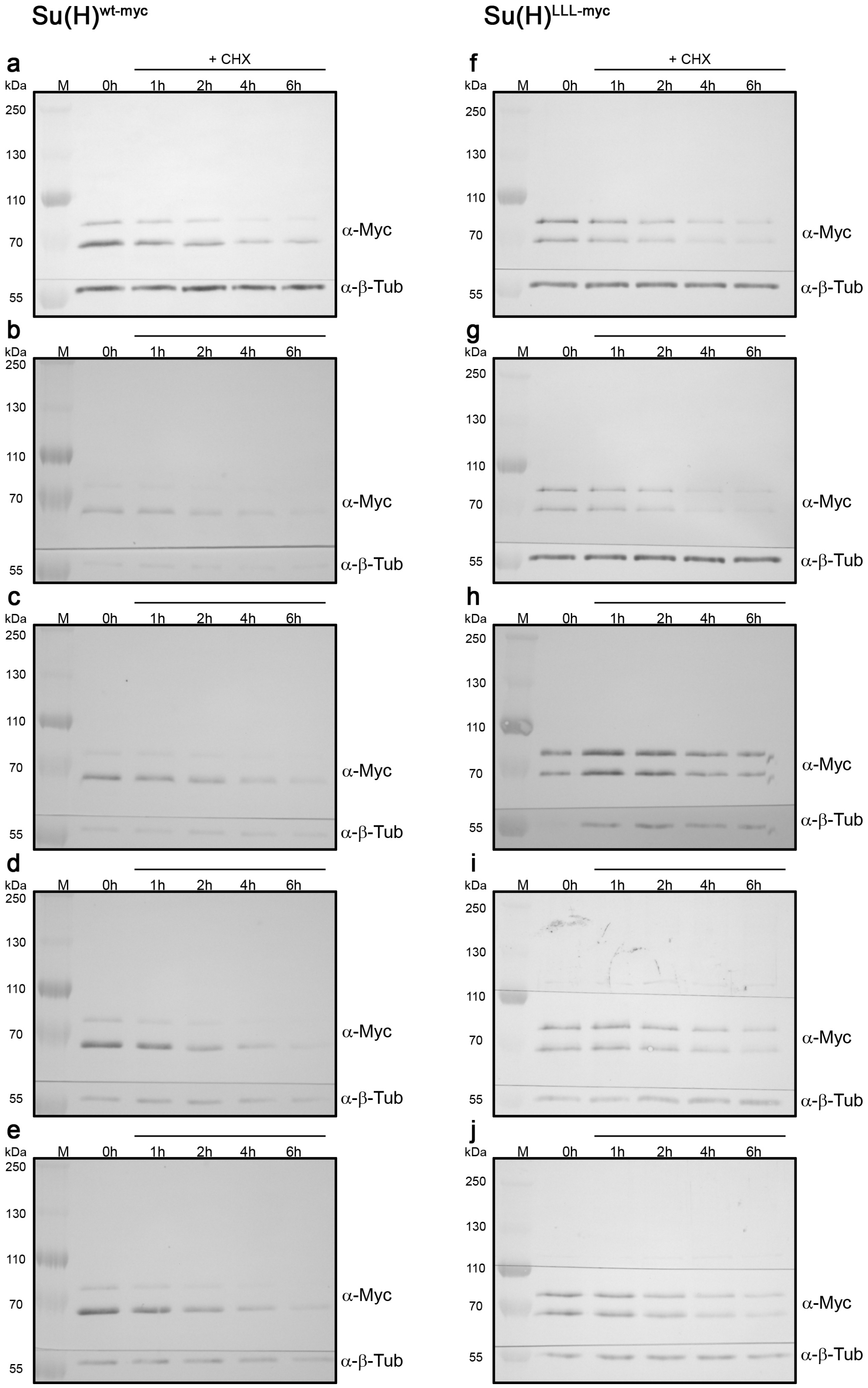

RBPJ^{KO} HeLa cells transfected with Su(H)$^{wt\text{-}myc}$ **(a-e)** or Su(H)$^{LLL\text{-}myc}$ **(f-j)**. Extracts prepared before cycloheximide (CHX) treatment (0h), and thereafter at the time indicated. Blots were cut to be probed simultaneously with anti-myc to detect Su(H), and anti-beta tubulin as internal standard. Size standard (M) in kDa.

Figure S4. Original Western blots used for the quantification of Su(H)[wt-myc] and Su(H)[LLL-myc] amounts in RBPJ[KO] HeLa cells in the presence of H.

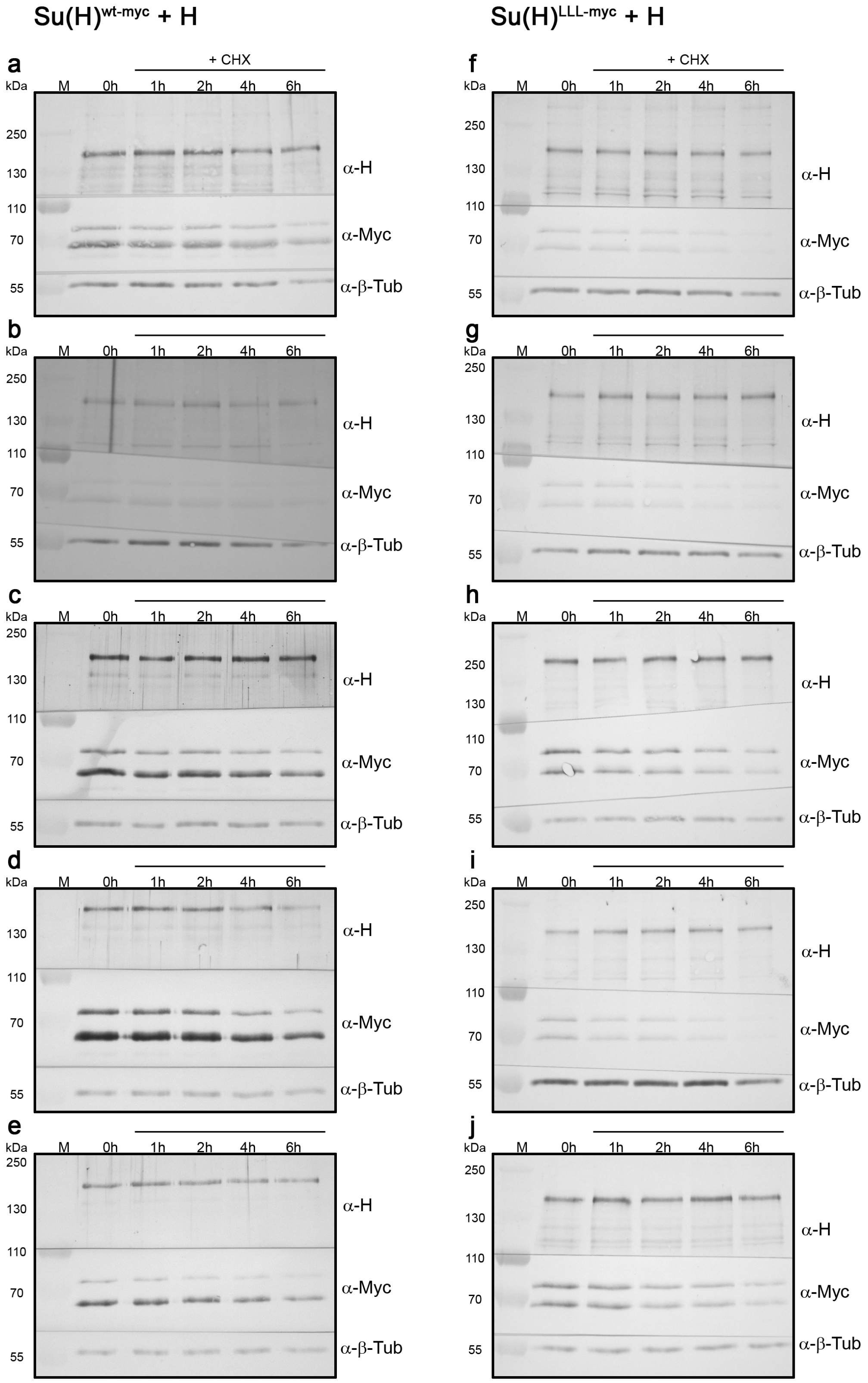

RBPJ[KO] HeLa cells co-transfected with Su(H)[wt-myc] **(a-e)** or Su(H)[LLL-myc] **(f-j)** as well as with H, detected with anti-myc for Su(H), anti-H, and anti-beta tubulin as internal standard. Extracts prepared before cycloheximide (CHX) treatment (0h), and thereafter at the time indicated. Blots were cut to be probed simultaneously with the respective antibodies. Size standard (M) in kDa.

Figure S5. Original Western blots used for the quantification of Su(H)$^{wt\text{-}myc}$ and Su(H)$^{LLL\text{-}myc}$ amounts in RBPJ^{KO} HeLa cells in the presence of Notch ICN^{RAMANK}.

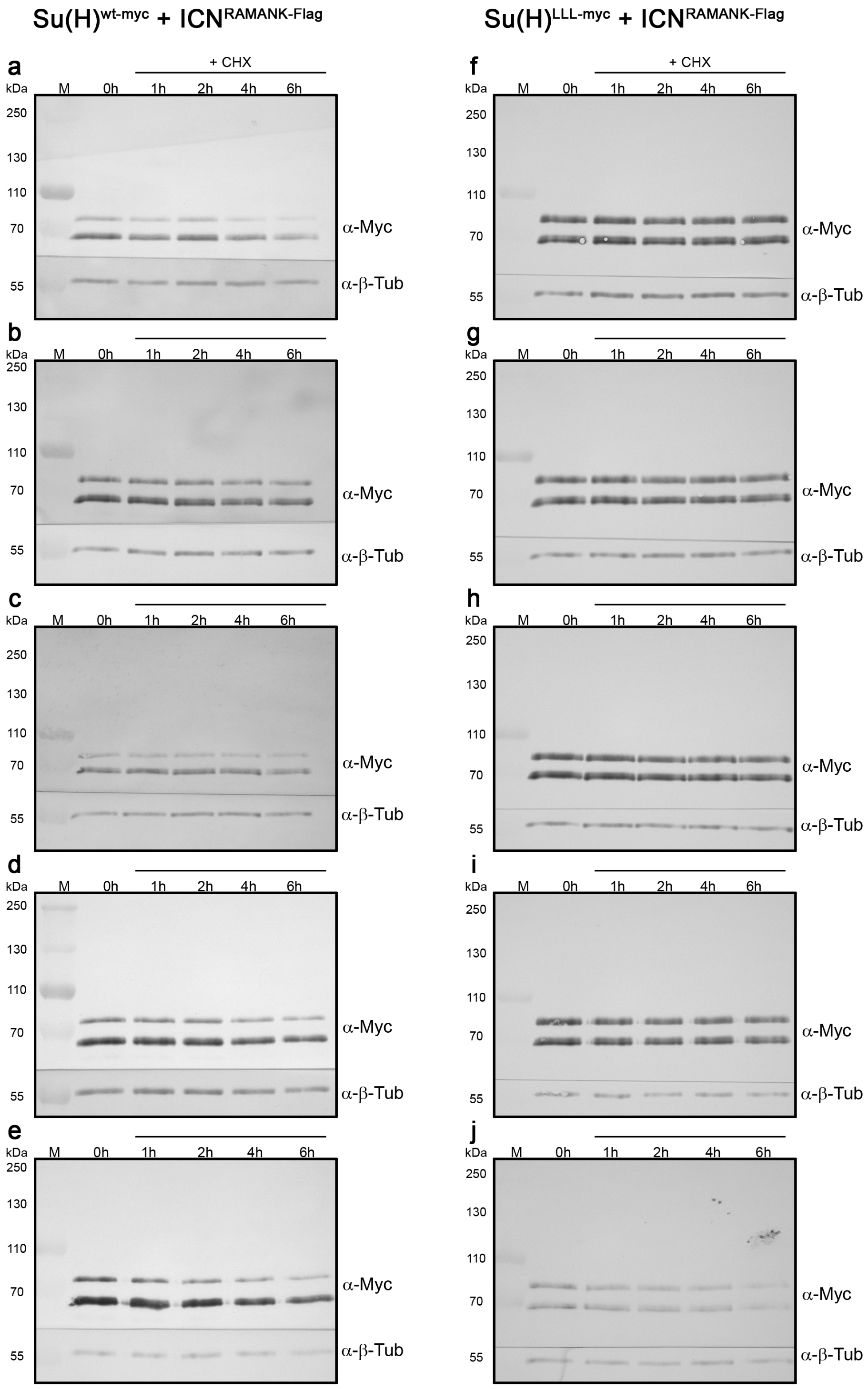

RBPJ^{KO} HeLa cells transfected with Su(H)$^{wt\text{-}myc}$ **(a-e)** or Su(H)$^{LLL\text{-}myc}$ **(f-j)** as well as with Notch ICN^{RAMANK}. Extracts prepared before cycloheximide (CHX) treatment (0h), and thereafter at the time indicated. Blots were cut to be probed simultaneously with anti-myc and anti-beta tubulin antibodies as internal standard. Size standard (M) in kDa.

Figure S6. Original Western blots used for the quantification of wild type human RBPJ and murine Rbpj amounts, respectively, in RBPJ^{KO} HeLa cells.

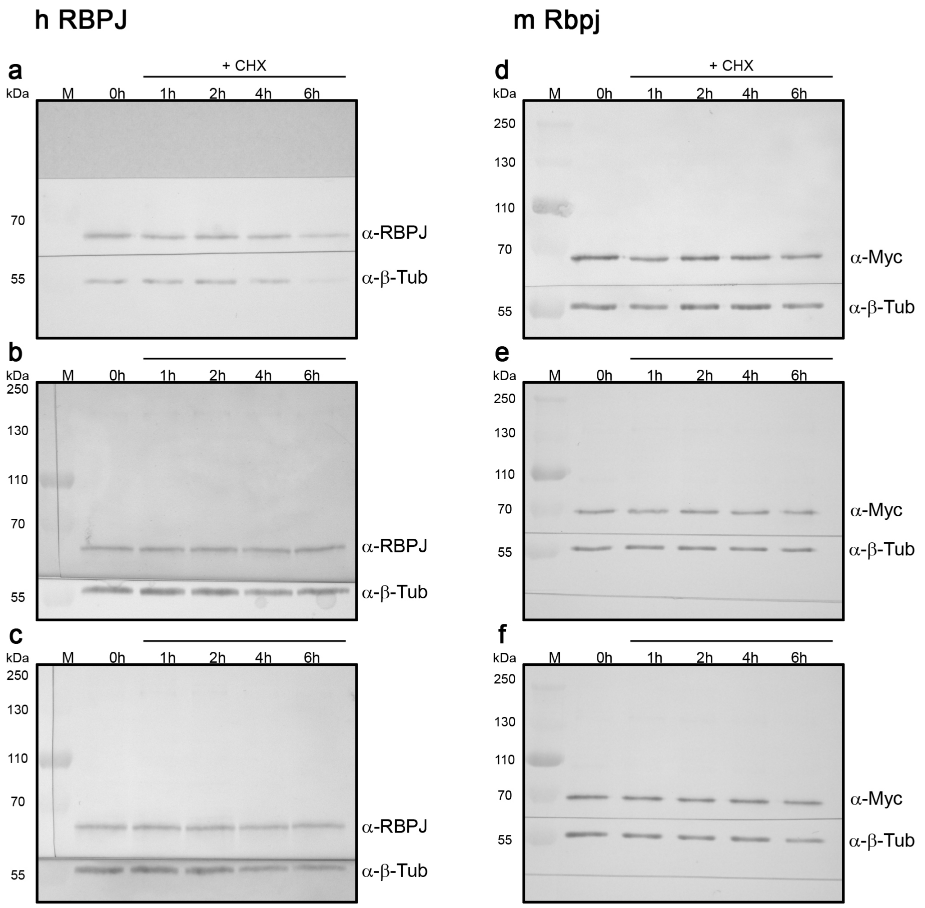

RBPJ^{KO} HeLa cells transfected with **(a-c)** human RBPJ and **(d-f)** myc-tagged murine Rbpj wild type constructs. Extracts prepared before cycloheximide (CHX) treatment (0h), and thereafter at the time indicated. Human RBPJ detected with anti-RBPSu(H) antibodies, murine Rbpj by the myc-tag. Blots were cut to be probed simultaneously with anti-beta-tubulin antibodies as internal standard. Size standard (M) in kDa.

Figure S7. Protein-interaction analyses of Rbpj mutant variants

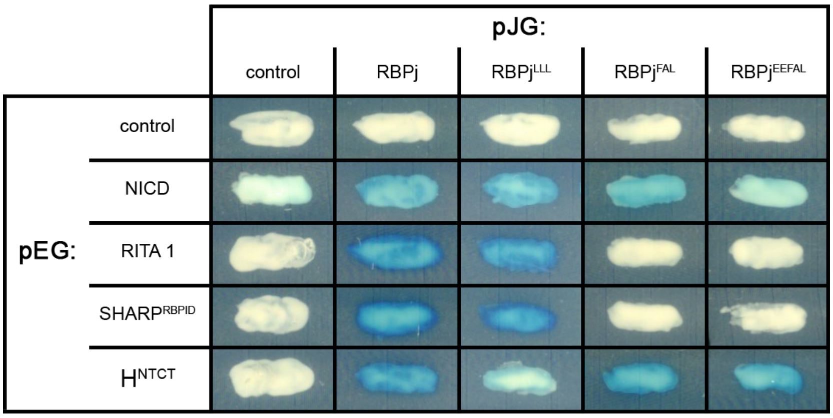

Yeast two-hybrid analyses to assay protein-protein interactions. Wild type murine Rbpj, $Rbpj^{FAL}$ and $Rbpj^{EEFAL}$, respectively, were provided in pJG 4-5 vector. *Drosophila* NICD and H-NTCT, human RITA1 and $SHARP^{RBPID}$, respectively, served as bait in pEG 202 vector. Empty vectors were used for control. Productive interactions induce blue coloration of yeast colonies resulting from the activation of the lacZ-reporter. The method is detailed in Golemis and Brent, 1997 [97].

Figure S8. Original Western blots used for the quantification of mutant murine Rbpj^{FAL} and Rbpj^{EEFAL} amounts, respectively, in RBPJ^{KO} HeLa cells

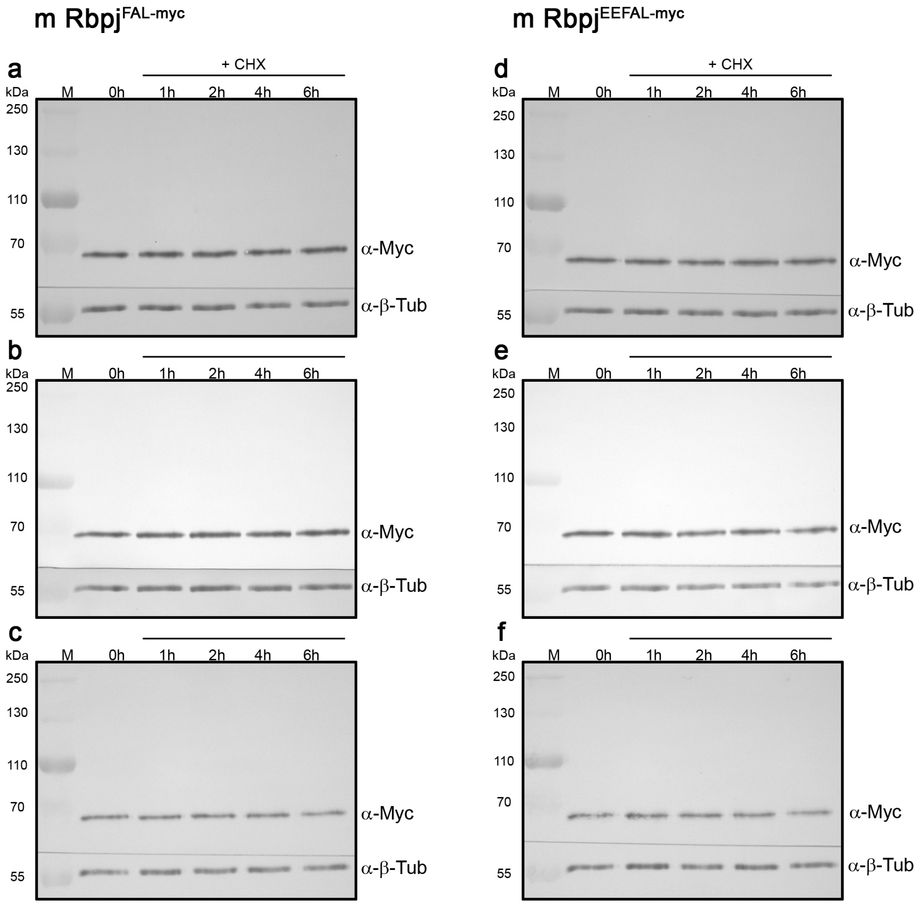

RBPJ^{KO} HeLa cells transfected with murine RBPJ^{FAL} **(a-c)** and Rbpj^{EEFAL} **(d-f)** mutant constructs, repesctively. Extracts prepared before cycloheximide (CHX) treatment (0h), and thereafter at the time indicated. The proteins were detected by the myc-tag; blots were cut to be probed simultaneously with anti-beta-tubulin antibodies as internal standard. Size standard (M) in kDa.

Table S1. List of primers used for mutagenesis

I. Adding a myc tag to Su(H) and Su(H)LLL cDNA
Amplify Su(H) with PCR primers to introduce a *Bam* HI site instead of the Su(H) Stop codon, yielding pBT-Su(H)*Bam after *Eco* RI/*Bam* HI digest and ligation.
SuH_EcoRI_up 5′ GAC ATG TGA ATT CAT GAA GAG CTA CAG CCA ATT TA 3′
SuH_BamHI_lo 5′ CAA TAA TCC TGC ACT CAG GAT CCG CCG CTA 3′

Annealed myc primers, ligated into *Bam* HI / *Xba* I digested Su(H)*Bam
5′ GAT CCG AGG AGC AGA AGC TGA TCT CGG AGG AGG ATC TGC TGT GAT 3′
5′ TCA GAT CAC AGC AGA TCC TCC TCC GAG ATC AGC TTC TGC TCC TCG 3′

II. Mutagenesis primers for Su(H)$^{K389/393A}$
Su(H)_K389A_up 5′-GCC GTG CCC CGC GGA GCC GAA CA-3′
Su(H)_K389A_lo 5′-GTG GCC TGG AAC TGT ATG ATT TTC-3′
Su(H)_K393A_up 5`-AGC CGA ACG CGG AGA TGA TCA ATG ACG GCG-3`
Su(H)_K393A_up 5`-CTG CAG GGC ACG GCG TGG CCT GGA ACT GTA T-3`

III. Mutagenesis primers for Su(H)^{K5A},
generated stepwise: first K389/393A (see above), second K103/104A, third K112A
Su(H)_K103/104A_up 5′ CAC TCG AGA CGC CAT GGA GAA GTA CAT GCG C 3′
Su(H)_K103/104A_lo 5′ AGC GCC GCC TCC TCG ATG TGT GGC CGG TAA G 3′
Su(H)_K112A_up 5′ TAC ATG CGC GAG CGC AAC GAC ATG GTC ATC 3′
Su(H)_K112A_lo 5′AGC TTC CAT GGC GTC TCG AGT GAG CGC CGC 3′

IV. Mutagenesis primers for Su(H)^{K5R},
generated stepwise: first K343/359R, second K200R, third K389/393R.
Su(H)K343/359Rup 5′ CGA TCC CGT TTC TCA GCT GCA CCG CTG CGC CTT CTA CAT GAA GGA TAC GG 3′
Su(H)K343/359Rlo 5′ TCG GCT TCC AGC AGG GCC ATC TGG CGG TCG ACC TTC CGG ATG ATC AGA CG 3′
Su(H)_ K200R_up 5′ ACG CTC TTC ATC TCG GAC TCG GAC AAG C 3′
Su(H)_ K200R_lo 5′ ACG GGC CGC ACA GTA CTG CTT GCC ATT GAG 3′
Su(H)_K389/393R_up 5′ GCG AGA TGA TCA ATG ACG GCG CCT GCT GGA CCA TCA TAT CCA C 3′
Su(H)_K389/393R_lo 5′ GAT TCG GCT CCC GCG GGC ACG GCG TGG CCT GGA ACT GTA TGA TTT T 3′

V. Rbpj subcloning into pBT-myc after *Bam* HI/*Xba* I digest
Rbpj_EcoRI_up 5′ GGT CAG CAG CAA AAT CAA GTG AAT CAT CTC AGT GCA ACT AAA 3′
Rbpj_BamHI_lo 5′ GGT GGG ATC CGG ACA CAA CTG TTG CTG TGG 3′

VI. Mutagenesis primers for Rbpj^{FAL} and Rbpj^{EEFAL},
generated stepwise: first F261A, second A284V, third L388A and finally E259/260A .
F261A_up 5′ GTT CGC GAT GGC TAC ATC CAT TAC G 3′
F261A_lo 5′ TGT GGC CTC CTC TCC TTC CGA CTC 3′
A284V_up 5′ CTG GCA TGG TGC TCC CAA GAT TGA TAA TTA GG 3′
A284V_lo 5′ TCA CAC TGC AGA CAA GCT TGA CAG TCT GC 3′
E259/260A_up 5′ CAC AGT TCG CGA TGG CTA CAT CCA TTA CGG 3′
E259/260A_lo 5′ GCC GCG GCT CCT TCC GAC TCG TCG TCG TC 3′
L388A_up 5′ TTC AGG CGA ATG GCG GCG GGG ACG TAG CAA TGC TTG AAC TTA C 3′
L388A_lo 5′ GAC TTT CTA CGA CAG GTA CCG GGG TGA CTG GGG CAA GGA C 3′
